# Construction and validation of a risk stratification model based on Lung-RADS^®^ v2022 and CT features for predicting the invasive pure ground-glass pulmonary nodules in China

**DOI:** 10.1186/s13244-025-01937-3

**Published:** 2025-03-23

**Authors:** Qingcheng Meng, Tong Liu, Hui Peng, Pengrui Gao, Wenda Chen, Mengjia Fang, Wentao Liu, Hong Ge, Renzhi Zhang, Xuejun Chen

**Affiliations:** 1 Department of Radiology, The Affiliated Cancer Hospital of Zhengzhou University & Henan Cancer Hospital, Zhengzhou, China; 2 Department of Radiotherapy, The Affiliated Cancer Hospital of Zhengzhou University & Henan Cancer Hospital, Zhengzhou, China; 3 Department of Radiology, National Cancer Center/National Clinical Research Center for Cancer/Cancer Hospital, Chinese Academy of Medical Sciences and Peking Union Medical College, Beijing, China

**Keywords:** Lung (ground glass nodule), Tomography (X-Ray computed), Lung reporting and data system, CT features

## Abstract

**Objectives:**

A novel risk stratification model based on Lung-RADS^®^ v2022 and CT features was constructed and validated for predicting invasive pure ground-glass nodules (pGGNs) in China.

**Methods:**

Five hundred and twenty-six patients with 572 pulmonary GGNs were prospectively enrolled and divided into training (*n* = 169) and validation (*n* = 403) sets. Utilising the Lung-RADS^®^ v2022 framework and the types of GGN-vessel relationships (GVR), a complementary Lung-RADS^®^ v2022 was established, and the pGGNs were reclassified from categories 2, 3 and 4x of Lung-RADS^®^ v2022 into 2, 3, 4a, 4b, and 4x of cLung-RADS^®^ v2022. The cutoff value of invasive pGGNs was defined as the cLung-RADS^®^ v2022 4a-4x. Evaluation metrics like recall rate, precision, F1 score, accuracy, Matthews correlation coefficient (MCC), and the area under the receiver operating characteristic curve (AUC) were employed to assess the utility of the cLung-RADS^®^ v2022.

**Results:**

In the training set, compared with the Lung-RADS 1.0, the AUC of Lung-RADS^®^ v2022 were decreased from 0.543 to 0.511 (*p*-value = 0.002), and compared to Lung-RADS 1.0 and Lung-RADS^®^ v2022, the cLung-RADS^®^ v2022 model exhibited the highest recall rate (94.9% vs 6.5%, 2.2%), MCC value (60.2% vs 5.4%, 6.3%), F1 score (92.5% vs 12.1%, 4.3%), accuracy (87.6% vs 23.1%, 19.5%), and AUC (0.718 vs 0.543, 0.511; *p*-value = 0.014, 0.0016) in diagnosing the invasiveness of pGGNs, and the similar performance was observed in the validation set.

**Conclusion:**

The cLung-RADS^®^ v2022 can effectively predict the invasiveness of pGGNs in real-world scenarios.

**Critical relevance statement:**

A complementary Lung-RADS^®^ v2022 based on the Lung-RADS^®^ v2022 and CT features can effectively predict the invasiveness of pulmonary pure ground-glass nodules and is applicable in clinical practice.

**Trial registration:**

Establishment and application of a multi-scale low-dose CT Lung cancer screening model based on modified lung-RADS1.1 and deep learning technology, 2022-KY-0137. Registered 24 January 2022. https://www.medicalresearch.org.cn/search/research/researchView?id=a97e67d8-1ee6-40fb-aab1-e6238dbd8f29.

**Key Points:**

- Lung-RADS^®^ v2022 delayed lung cancer diagnosis for nodules appearing as pGGNs.
- Lung-RADS^®^ v2022 showed lower accuracy and AUC than Lung-RADS 1.0.
- cLung-RADS^®^ v2022 model effectively predicts the invasiveness of pulmonary pGGNs.

**Graphical Abstract:**

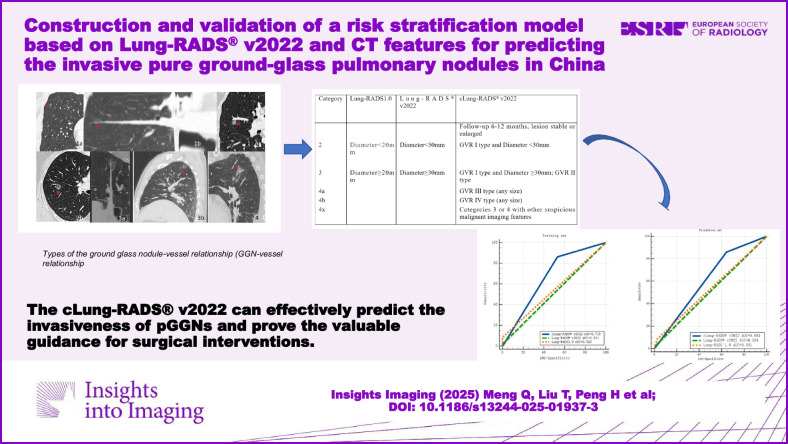

## Introduction

Lung cancer (LC) is the leading cause of cancer incidence and mortality in China [1]. Low-dose computed tomography (CT) screening for LC is a promising strategy for improving the detection rate of early LC and reducing the associated mortality [2, 3]. Therefore, the detection rate of pulmonary pure ground-glass nodules (pGGNs) has increased dramatically because of the widespread use of CT screening programmes for LC detection [4]. The spectrum of diseases associated with pGGNs primarily includes pulmonary haemorrhage, fibrosis, atypical adenomatous hyperplasia, in situ carcinoma, minimally invasive adenocarcinoma, and invasive adenocarcinoma. To reduce the false positive rate of LCs, the American College of Radiology (ACR) introduced the Lung Imaging Reporting and Data System (Lung-RADS), which stratifies management based on nodule size or volume. However, this system offers low-risk stratification for pGGNs, potentially delaying the detection of early-stage LC and reducing the benefits of screening [5]. Furthermore, in May 2021, the World Health Organization (WHO) issued the fifth edition of the classification of thoracic tumours, categorising atypical adenomatous hyperplasia and in situ carcinoma as adenomatous precursor lesions [6]. This addition complicates CT imaging diagnoses in the screening programme. Most pGGNs represent early-stage LC, with a postoperative 5-year survival rate approaching 100% [7, 8]; precise preoperative risk stratification is crucial to determine the optimal timing of surgery, the extent of surgical resection, and the necessity of lymph node dissection [9–11]. In November 2022, the ACR updated its guidelines using the Lung-RADS^®^ v2022 [12], further standardising CT reports for LC screening populations to aid clinical decisions. However, the 4x category in this system lacks clear indicators related to imaging features, and the cutoff value of size with more than and equal to 30 mm delayed the diagnosis of early LC. This study aims to develop a novel risk stratification model-based Lung-RADS^®^ v2022 and CT features, evaluating its practical value in assessing the invasiveness of pGGNs and guiding surgical interventions in real-world scenarios.

## Materials and methods

This study was approved by the Ethics Committee of the Affiliated Cancer Hospital of Zhengzhou University and Henan Cancer Hospital (ethical number: 2022-KY-0137) and complied with its ethical standards.

### Patient characteristics

**Fig. 1:**
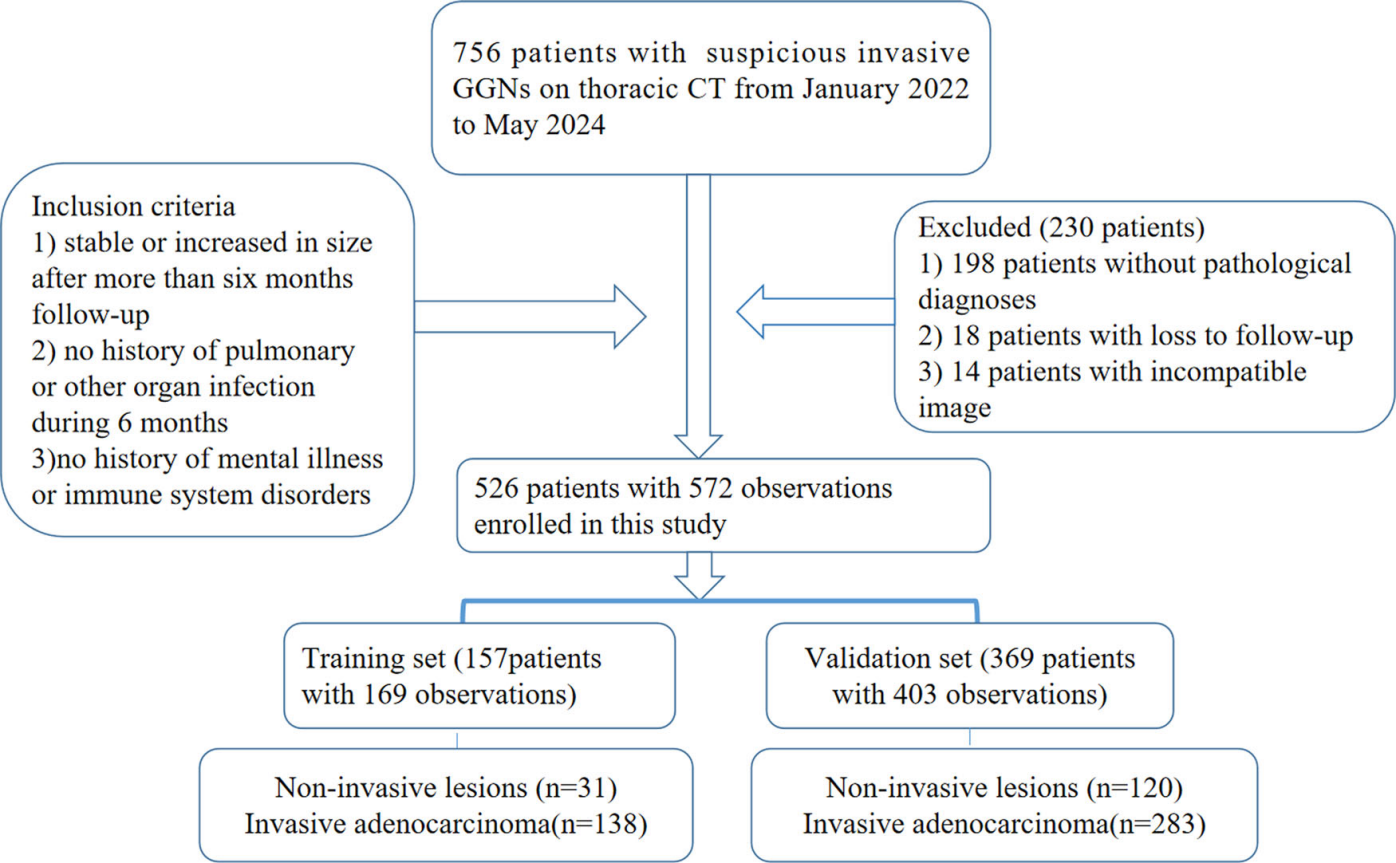
Flowchart of the patient selection procedure

Patients with solitary or multiple pGGNs on CT images were prospectively recruited at the Affiliated Cancer Hospital of Zhengzhou University and Henan Cancer Hospital between January 2022 and May 2024. The inclusion criteria were as follows: (1) presence of solitary or multiple pulmonary pGGNs with clear borders on CT images, (2) stable or increased diameter of pGGNs after more than 3 months of follow-up, (3) no history of pulmonary or other organ infection for 6 months, and (4) no history of mental illness or immune system disorders. The exclusion criteria included lack of postoperative pathology, loss to follow-up, and incomplete or suboptimal CT images. A total of 157 patients with 169 pGGNs enrolled from January to December 2022 were designated as the training set, while 369 patients with 403 pGGNs enrolled from January 2023 to May 2024 were allocated to the validation set. Of these, 307 patients with 318 GGNs were previously reported [13]. According to the fifth edition of the WHO classification of thoracic tumours [6], minimally invasive adenocarcinoma and invasive adenocarcinoma were designated as invasive lesions requiring surgical intervention, and the inflammation, benign lesions, and adenomatous precursor lesions were grouped as noninvasive lesions, subject to regular follow-up. The subject selection process is illustrated in Fig. 1.

### CT scan

All patients underwent low-dose chest CT scans one week before the operation using either a LightSpeed-16 scanner (GE) or an iCT-256 scanner (Siemens). The scan parameters included a tube voltage of 120 kVp, tube current ranging from 50 to 200 mA, matrix size of 512 × 512, collimation of 128 × 0.625 mm or 16 × 1.25 mm, rotation time of 0.5 s, pitch of 0.8 or 1.02, and reconstruction layer thickness of 1.25 mm. A nonionic contrast agent was administered for the multiphase-enhanced scanning. The scanning range extended from the lung apex to the costophrenic angle and encompassed the entire lung field. Scans were acquired at the end of full inhalation with breath-holding as instructed by trained personnel.

### CT images analysis

**Fig. 2:**
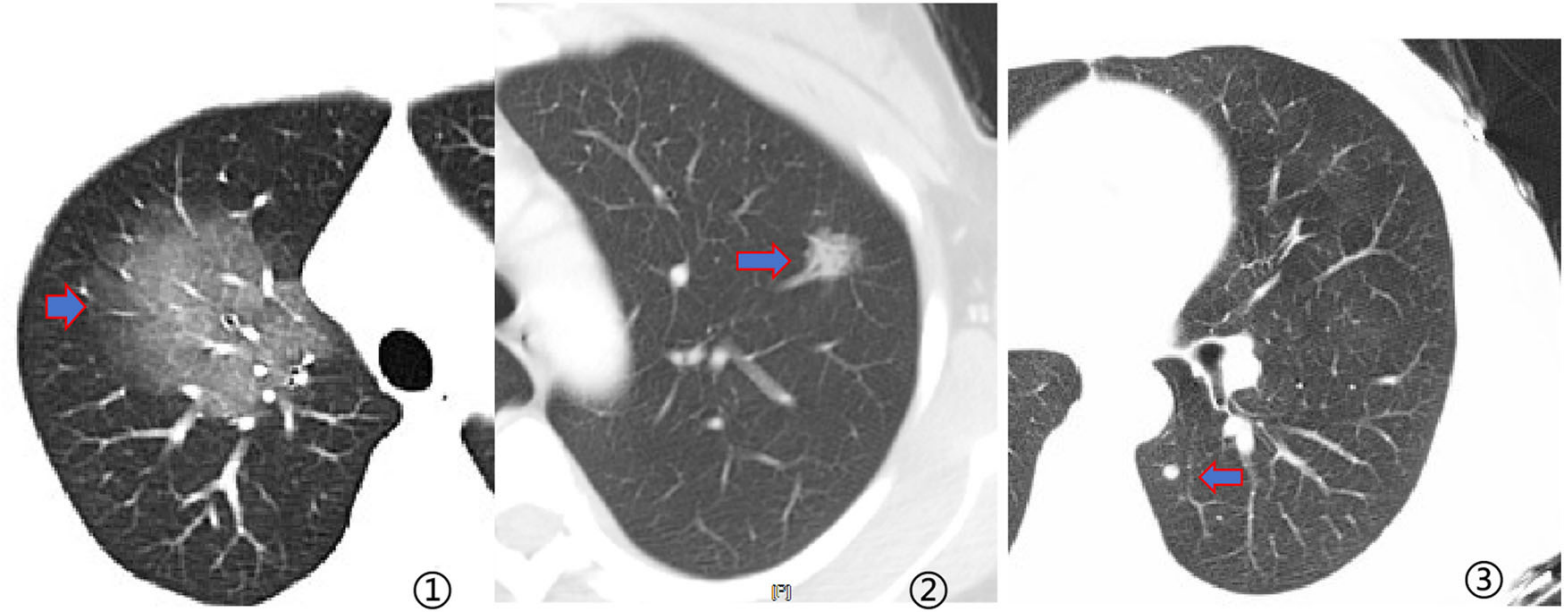
Types of border of lesions. ①: Blurry border of GGNs; ②: clear border of GGNs; and ③: sharp border of lesions

Standard lung windows (window width/window level: 1600–2000 HU/600–700 HU) and mediastinal windows (350–380/1015 HU) were used for observation. Multiplanar reconstructions (sagittal, coronal, and axial) and maximum intensity projections (MIP) were used to display morphological features and relationships with adjacent structures. To reflect the true size of the nodule, the mean diameter was determined by calculating the average of the maximum diameter and its perpendicular minor diameter on lung-window CT images in any plane. Initially, to diagnose the clear border, three types of pulmonary-nodule borders—blurry, clear, and sharp—are shown in Fig. 2. Two chest imaging experts achieved a consensus in recording the CT features of the lesions based on the thin-slice CT image sequence.

### Design of complementary Lung-RADS^®^ v2022 model based on CT features and Interpretation of positive nodules

**Fig. 3:**
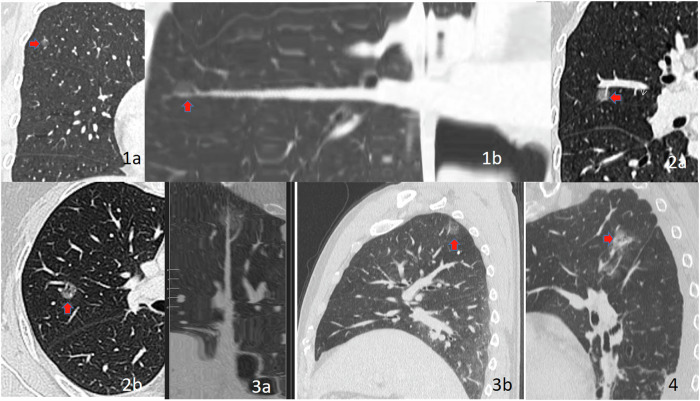
Types of the ground glass nodule-vessel relationship (GGN-vessel relationship, GVR). GVR I type: vessels passing beside the nodule (**1a**, **1b**); GVR II type: vessels passing through the nodule without thickening or distortion (**2a**, **2b**); GVR III type: vessels passing through the nodule with localised thickening (**3a**, **3b**); GVR IV type: increased, thickened, twisted, and convoluted vessels (**4**)

**Fig. 4:**
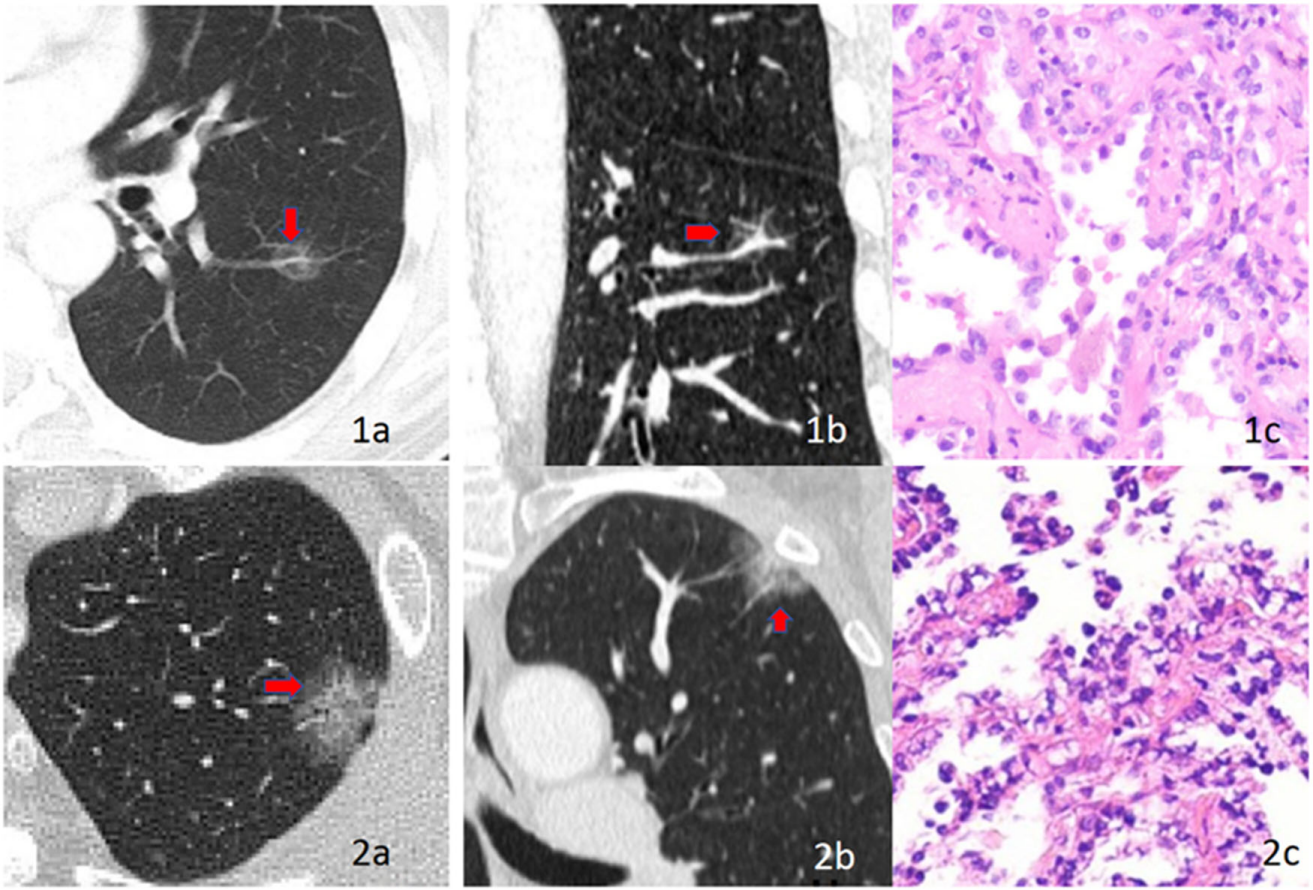
Stratification of GGNs with cLung-RADS^®^ v2022 category. **1a**–**c** Male, 49 years old, pure GGN of low lobe of left lung detected after lung cancer screening, the size is 15 mm and the G-V-R is III type, so the cLung-RADS^®^ v2022 category is 4a, Pathology: Microinvasive Adenocarcinoma. **2a**–**c** Female, 58 years old, the size of GGN in the upper lobe of left lung is 35 mm and the G-V-R type is IV, so the cLung-RADS^®^ v2022 category is 4x, Pathology: Invasive Adenocarcinoma. cLung-RADS^®^ v2022, Complementary Lung Reporting and Data System version 2022; G-V-R, GGN-vessel relationship

For GGNs with stable or enlarged sizes after a 3- to 6-month follow-up CT scan, maximum intensity projection and multiplanar reconstruction are recommended to evaluate the supply of blood vessels. Based on the ground-glass nodule-vessel relationship (GGN-vessel relationship, GVR), four types were delineated [7], as shown in Fig. 3: GVR I type (vessels passing beside the nodule), GVR II type (vessels passing through the nodule without thickening or distortion), GVR III type (vessels passing through the nodule with localised thickening), and GVR IV type (increased, thickened, twisted, and convoluted vessels). Based on the above, a novel cLung-RADS^®^ v2022 model for pGGNs was developed based on the GVR types. The classification criteria were as follows: GVR I type with a diameter < 30 mm was categorised as cLung-RADS^®^ v2022 category 2, GVR I type with a diameter ≥ 30 mm or GVR II type was categorised as cLung-RADS^®^ v2022 category 3; any size nodule with GVR III type was categorised as cLung-RADS^®^ v2022 category 4a; nodule with GVR IV type, regardless of size, was categorised as cLung-RADS^®^ v2022 category 4b; and category 3 or 4 with other suspicious malignant imaging features were defined as cLung-RADS^®^ v2022 category 4x, as shown in Table 1 and Fig. 4. Nodules classified as cLung-RADS^®^ v2022 categories 4a-4x were defined as invasive pGGNs requiring surgical intervention.

**Table 1:** Comparison of classification criteria for pulmonary pure ground-glass nodules in Lung-RADS1.0, Lung-RADS^®^ v2022, and cLung-RADS^®^ v2022

Category	Lung-RADS1.0	Lung-RADS^®^ v2022	cLung-RADS^®^ v2022
			Follow-up 6–12 months, lesion stable or enlarged
2	Diameter < 20 mm	Diameter < 30 mm	GVR I type and diameter < 30 mm
3	Diameter ≥ 20 mm	Diameter ≥ 30 mm	GVR I type and diameter ≥ 30 mm; GVR II type
4a			GVR III type (any size)
4b			GVR IV type (any size)
4x			Categories 3 or 4 with other suspicious malignant imaging features

*GVR* nodule-vessel relationship type, *Lung-RADS* Lung Reporting and Data System, *cLung-RADS*^*®*^ *v2022* Complementary Lung Reporting and Data System version 2022

### Statistical analysis

Statistical analyses were conducted using SPSS version 24.0. Descriptive statistics for continuous variables are presented as mean ± standard deviation and independent *t*-tests were used for comparisons. Categorical data are expressed as percentages, and comparisons were made using the chi-square test. Interobserver consistency among the different readers for Lung-RADS classification was assessed using interclass correlation coefficients. The performance of various Lung-RADS classification models was evaluated using metrics such as (1) recall rate (${Recall}=\frac{{TP}}{{TP}+{FN}}$); (2) precision (${Precision}=\frac{{TP}}{{{{\rm{TP}}}}+{{{\rm{FP}}}}}$); (3) accuracy (${ACC}=\frac{{TP}+{TN}}{{TP}+{FP}+{TN}+{FN}}$); (4) F1 score ($F1=\frac{2\times {{{\rm{Precision}}}}\times {{{\rm{Recall}}}}}{{{{\rm{Precision}}}}+{{{\rm{Recall}}}}}$), (5) weighted average F1 score ($F1{weighted}=\frac{(1+{{{{\rm{\beta }}}}}^{2})\times {{{\rm{Precision}}}}\times {{{\rm{Recall}}}}}{({{{{\rm{\beta }}}}}^{2}\times {{{\rm{Precision}}}})\,+{{{\rm{Recall}}}}}$), and (6) Matthews correlation coefficient (${MCC}=\frac{{TP}\times {{{\rm{TN}}}}-{{{\rm{FP}}}}\times {{{\rm{FN}}}}}{\sqrt{({TP}+{FP})({TP}+{FN})({TN}+{FP})({TN}+{FN})}}$). In these metrics, true positives (TP) represent pGGN diagnosed as invasive by the risk model and confirmed as malignant by pathology; false positives (FP) indicate pGGNs diagnosed as invasive by the risk model but confirmed as benign by pathology; true negatives (TN) indicate pGGNs diagnosed as benign by the risk model and confirmed as benign by pathology; false negatives (FN) indicate pGGNs diagnosed as benign by the risk model but confirmed as malignant by pathology. To attenuate the influence of false negatives, we set β = 0.5 to calculate F1_weighted_. The overall performance was further assessed by the area under the receiver operating characteristic curve (AUC), and the DeLong test was used to assess the statistical significance of the differences among the three models, with a significance level set at *p* < 0.05.

## Results

### Dataset characteristics

In total, 526 patients with 572 pulmonary ground-glass nodules (pGGNs) were included in this study. The distribution of pGGNs was as follows: 487 patients with solitary pGGNs, 32 patients with two pGGNs, and 7 patients with three pGGNs. The mean follow-up duration for patients in the training set was 43.6 ± 11.3 months, while the validation set had an average follow-up of approximately 43.5 ± 12.1 months. The pathological spectrum of all pGGNs comprised invasive adenocarcinomas (*n* = 421) and noninvasive lesions (*n* = 151), with 39 inflammatory lesions, 3 sclerosing pneumocytomas, and 109 adenomatous precursor lesions. The distribution of the GVR types in the training set was as follows: 15 pGGNs of type I, 19 of type II, 19 of type III, and 116 of type IV. In contrast, the validation set included 35 type I, 41 type II, 51 type III, and 276 type IV pGGNs. No statistically significant differences were observed between the training and validation sets in terms of sex, age, family history of carcinoma, clinical symptoms, chronic obstructive pulmonary disease, follow-up period, adherence to medical orders, distribution of pGGNs, GVR type of pGGNs, or lung disease spectrum (all *p* < 0.05; Table 2).

**Table 2:** Clinical characteristics of patients between training set and validation set (means ± standard deviations; *n* (%))

Characteristics	Training set	Validation set	*p*-value
Gender			0.579
Male	58	127	
Female	99	242	
Age (years)	57.5 ± 9.1	56.2 ± 9.7	0.158
Family history of carcinoma			0.687
Yes	6	17	
No	151	352	
Clinical symptoms			0.231
Yes	27	49	
No	129	320	
Chronic obstructive pulmonary disease			0.987
Yes	11	26	
No	146	343	
Period of follow-up (months)	43.6 ± 11.3	43.5 ± 12.1	0.990
Compliance with medical orders			0.208
Yes	101	258	
No	56	111	
Distribution of nodules in patients			0.663
One	146	340	
Two	10	23	
Three	1	6	
G-V-R type			0.954
I	15	35	
II	19	41	
III	19	51	
IV	116	276	
Size of pGGNs (mm)	13.8 ± 6.4	13.5 ± 6.0	0.593
Lung adenocarcinoma spectrum			0.459
Noninvasive lesions (including benign lesion and adenomatous precursor lesions)	49	120	
Invasive adenocarcinoma	120	283	

*G-V-R* GGN-vessel relationship, *pGGNs* pure ground-glass nodules, *CT* computed tomography

### Interobserver consistency among three readers

A subset of 100 patients with 100 pGGNs were randomly selected for stratified management by three experienced radiologists on chest imaging with varying levels of experience (6, 7, and 9 years) using Lung-RADS 1.0, Lung-RADS^®^ v2022, and cLung-RADS^®^ v2022. The interclass correlation coefficients between the different observers were 1.00, 1.00, and 0.974, indicating excellent consistency (Table 3).

**Table 3:** Consistency among different Lung-RADS systems across observers

Lung-RADS system	Classification	Observer1	Observer2	Observer3	ICC	*p*-value
Lung-RADS 1.0					1.00	0.00
	2	91	92	92		
	3	7	6	7		
	4x	2	2	1		
Lung-RADS^®^ v2022					1.00	0.00
	2	95	94	93		
	3	3	3	5		
	4x	2	3	2		
cLung-RADS^®^ v2022					0.974	0.00
	2	27	27	27		
	3	15	16	14		
	4a	36	38	35		
	4b	19	13	19		
	4x	3	6	5		

*Lung-RADS* Lung Reporting and Data System, *cLung-RADS*^*®*^ *v2022* Complementary Lung Reporting and Data System version 2022, *ICC* intra-class correlation coefficient

### Value between the Lung-RADS 1.0 and Lung-RADS^®^ v2022 in diagnosing the invasiveness of pGGNs

In the training set, compared with the Lung-RADS 1.0, the recall rate, F1 score, F1_weighted_, accuracy and AUC of Lung-RADS^®^ v2022 were decreased from 2.2% to 6.5%, 4.3% to 12.1%, 10.1% to 25.2%, 19.5% to 23.1%, and 0.511 to 0.543 (*p*-value = 0.002). Similarly, in the validation set, Lung-RADS 1.0 outperformed Lung-RADS^®^ v2022 in recall rate (9.5% to 1.8%), F1 score (17.2% to 3.5%), F1weighted (36% to 31%), and AUC (0.531 to 0.509), with a *p*-value of 0.0047, highlighting the necessity for revising Lung-RADS^®^ v2022.

### Diagnostic value of different models for invasive pGGNs

**Fig. 5:**
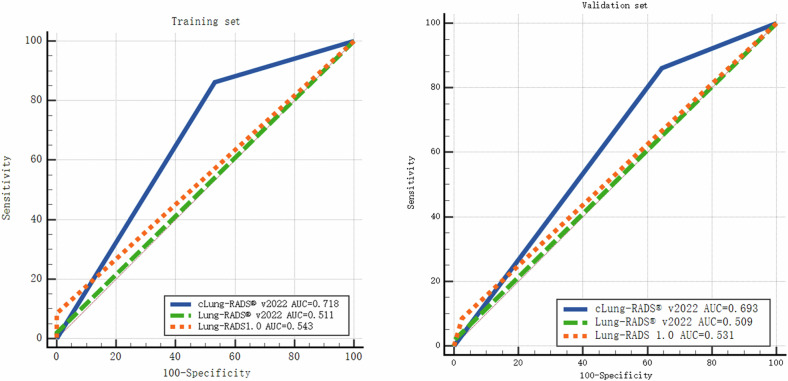
Comparison of the AUCs of Lung-RADS 1.0, Lung-RADS^®^ v2022 and cLung-RADS^®^ v2022 in the training set and validation set. In the training set, the AUCs of Lung-RADS 1.0, Lung-RADS^®^ v2022 and cLung-RADS^®^ v2022 were 0.543 (95% CI: 0.465–0.620), 0.511 (95% CI: 0.433–0.588) and 0.718 (95% CI: 0.628–0.819), respectively, with *p*-value = 0.014, 0.0016. In the validation set, the AUCs of Lung-RADS 1.0, Lung-RADS^®^ v2022 and cLung-RADS^®^ v2022 were 0.531 (95% CI: 0.481–0.580), 0.509 (95% CI: 0.459–0.559) and 0.693 (95% CI: 0.594–0.773) respectively, with *p*-value = 0.0036, 0.0003. AUC, area under receiver operating characteristic curve; Lung-RADS, Lung Reporting and Data System; cLung-RADS^®^ v2022, Complementary Lung Reporting and Data System version 2022

In the training set, compared with the Lung-RADS 1.0 and v2022, the cLung-RADS^®^ v2022 model demonstrated significantly improved metrics for the diagnosis of invasive pGGNs, including recall rate (94.9%, 6.5% and 2.2%), MCC values (60.2%, 5.4%, and 6.3%), F1 score (92.5%, 12.1%, and 4.3%), F1_weighted_ (91.2%, 25.2%, 10.1%), and ACC (87.6%, 23.1%, 19.5%). The AUC for the cLung-RADS^®^ v2022 model reached 0.718, surpassing the AUC for Lung-RADS 1.0 (0.543) and Lung-RADS^®^ v2022 (0.511), with *p*-values of 0.014 and 0.0016, respectively. To further validate the efficacy of the cLung-RADS^®^ v2022 model, a larger validation set (*n* = 403) was used to validate. The model exhibited a recall rate of 90.8%, MCC value of 50.6%, F1 score of 86.7%, F1_weighted_ of 89.1%, and ACC of 80.4%, still outperforming the comparable data from Lung-RADS 1.0 and Lung-RADS^®^ v2022. In the validation set, the AUC for the cLung-RADS^®^ v2022 model was 0.693, surpassing Lung-RADS 1.0 (AUC = 0.531) and Lung-RADS^®^ v2022 (AUC = 0.509), *p*-value = 0.0036 and 0.0003, respectively. This indicates the capability of the model to selectively identify pGGNs requiring timely surgical intervention, as shown in Table 4 and Fig. 5.

**Table 4:** Value of Lung-RADS1.0, Lung-RADS^®^ v2022, and cLung-RADS^®^ v2022 in diagnosing invasive pure ground-glass nodules

	Training set			Validation set		
	Lung-RADS1.0	Lung-RADS^®^ v2022	cLung-RADS^®^ v2022	Lung-RADS1.0	Lung-RADS^®^ v2022	cLung-RADS^®^ v2022
TP	9	3	131	27	5	257
FP	1	0	14	2	0	53
FN	129	135	7	256	278	26
TN	30	30	17	118	120	67
Recall, %	6.5	2.2	94.9	9.5	1.8	90.8
Precision, %	90.0	100	90.3	93.1	100	82.9
MCC, %	5.4	6.3	60.2	13.9	7.3	50.6
F1 score (%)	12.1	4.3	92.5	17.2	3.5	86.7
F1_weighted_ (%)	25.2	10.1	91.2	33.7	8.4	89.1
Accuracy, %	23.1	19.5	87.6	36.0	31.0	80.4
AUC	0.543	0.511	0.718	0.531	0.509	0.693

*Lung-RADS* Lung Reporting and Data System, *cLung-RADS*^*®*^ *v2022* Complementary Lung Reporting and Data System version 2022, *MCC* Matthews Correlation Coefficient, *ACC* accuracy, *AUC* area under the curve

## Discussion

In this study, we introduce a novel cLung-RADS^®^ v2022 model designed to predict invasive lesions that manifest as pGGNs on CT images. Our model was trained and validated using data from patients with lung GGNs and exhibited excellent performance in identifying invasive lesions with high accuracy, recall rate, and AUC in both the training and validation sets. Our findings suggest that the cLung-RADS^®^ v2022 holds significant potential value for LC risk prediction and decision support in the management of pulmonary pGGNs. By effectively ruling out unnecessary imaging and invasive procedures through the model’s high F1weighted scores (91.2% and 89.1% for training and validation, respectively) and AUC (0.718 and 0.693, respectively), we could potentially reduce the burden of excessive workups on a considerable number of patients.

The increasing incidence of LC in non-smokers and females in Asian populations, often presenting as pGGNs, has drawn significant attention [13–15], In a multicentre study of pGGNs by our team, the percentage of invasive pGGNs was approximately 83.06% [16]. As the Lung-RADS^®^ v2022 risk stratification system proposed by the American College of Radiology is based on Western populations at high risk for LC, the diameter cutoff for pGGN was revised from the 20 mm of Lung-RADS 1.0 to the 30 mm of the Lung-RADS^®^ v2022. However, our study observed a decrease in the AUC for stratification of the invasive pGGNs from 0.543 for the Lung-RADS 1.0 to 0.511 for Lung-RADS^®^ v2022 in the training set (*p*-value = 0.002), which could delay the early diagnosis of invasive pGGNs and impact the clinical decision-making. Therefore, we made targeted modifications to the lung nodule risk stratification model based on Lung-RADS^®^ v2022, incorporating CT features value [17]. To assess the practicality of cLung-RADS^®^ v2022 for stratified management of GGNs, we initially randomly selected 100 pGGNs and three chest imaging experts independently were selected to evaluate these nodules in a blinded manner, and the consistency coefficient was used to assess the consistency of results among the three experts. The results indicated excellent consistency among the three experts.

Recently, with the rapid advancement of artificial intelligence in the medical field, radiomics and deep learning technologies have been explored to some extent for the risk stratification management of pGGNs. Sun et al [18] utilised radiomics in conjunction with CT signs to predict the invasiveness of pGGNs and achieved the expected results. However, the heterogeneity of samples in different radiomic research projects presents challenges in terms of repeatability and clinical applications. Hu et al [19] used a combination of deep neural networks and radiomics for the stratified management of malignant pGGNs, achieving an accuracy of 76.5%, F1 score of 84.6%, and Matthews correlation coefficient of 43.6%. Considering the complexity of the medical field and the ongoing need for iterations and improvements in artificial intelligence research, there is an urgent demand for simple, operable, and stable predictive models of pGGNs. In light of this, our study developed the cLung-RADS^®^ v2022 model based on CT features, focusing on vascular changes in ground-glass nodules typical of LC. To evaluate the real-world application of the cLung-RADS^®^ v2022 model for stratified management of pGGNs, parameters such as recall rate, precision, F1 score, Matthews correlation coefficient, accuracy, and area under the curve were used for validation. Furthermore, to validate real-world efficacy, the inclusion ratio of nodules in the validation set of this study was higher than that in the training set. Compared with the results of Hu et al’s study [19], our cLung-RADS^®^ v2022 model achieved higher accuracy (87.6%), F1_weighted_ (91.2%) and recall rate (94.9%) in the training set, and the similar high diagnostic performance was achieved in the validation set, all of which did benefit for the LC screening in clinical. Furthermore, the AUC of cLung-RADS^®^ v2022 among the two datasets were higher than those of Lung-RADS 1.0 or Lung-RADS^®^ v2022, all *p* < 0.05, which showed excellent performance in diagnosing invasive GGNs in this dataset.

Our study does have some limitations. First, it was a single-centre study, which may have been affected by design bias. The small sample size in the noninvasive group was another limitation of this study, which may have affected the diagnostic power. Finally, the cLung-RADS^®^ v2022 model does not eliminate the risk of overtreatment because some invasive pGGNs may be indolent and clinically insignificant, potentially affecting a patient’s quality of life. However, Henschke et al [20] found that almost all of diagnosed but untreated stage IA non-small cell LC, as small as 10 mm in diameter, have a malignant natural course and are fatal if not treated. Therefore, further evaluation of the effectiveness of cLung-RADS^®^ v2022, with more pGGNs cases and several centres in the world, is warranted.

## Conclusion

In summary, the cLung-RADS^®^ v2022 model, based on CT features and designed for risk stratification of pGGNs, has excellent diagnostic efficacy and shows promise in guiding timely surgical intervention of pGGNs in clinical practice. The ability of the model to differentiate between invasive and noninvasive pGGNs with high precision could significantly enhance the management of patients with pulmonary pGGNs, potentially reducing the incidence of unnecessary procedures and ensuring the prompt treatment of invasive lesions.

## Data Availability

The datasets used and/or analysed during the current study are available from the corresponding author upon reasonable request.

## Abbreviations

ACC: Accuracy
AUC: Area under the ROC curve
cLung-RADS^®^ v2022: Complementary Lung Imaging Reporting and Data System, version 2022
F1_weighted_: Weighted average F1 score
LC: Lung cancer
Lung-RADS: Lung Imaging Reporting and Data System
MCC: Matthews correlation coefficient
pGGN: Pure ground-glass nodule
